# Behçet’s Disease in a Patient With Non-Hodgkin Lymphoma: A Case of Genital Ulceration Resembling a Sexually Transmitted Disease

**DOI:** 10.7759/cureus.91050

**Published:** 2025-08-26

**Authors:** Nabila Arkania, Rizky Rahma Wijayanti, Fatima Kus Megawati, Rizka Fauziyah, Sri Awalia Febriana

**Affiliations:** 1 Department of Dermatovenereology, Faculty of Medicine, Public Health, and Nursing, Gadjah Mada University, Special Region of Yogyakarta, IDN

**Keywords:** behçet’s disease, genital ulcers, immunological comorbidities, multisystem inflammatory disease, non-hodgkin lymphoma (nhl)

## Abstract

The symptoms of Behçet’s disease (BD), a chronic, multisystem inflammatory disease, include skin lesions, uveitis, oral and vaginal mucosal ulcers, and involvement of other organ systems. Genital ulcers are one of the main manifestations of BD and can mimic sexually transmitted infections (STIs). It can be difficult to diagnose BD, particularly in patients who have immunological comorbidities such as non-Hodgkin lymphoma (NHL). This case report highlights the importance of a comprehensive diagnostic approach for atypical vaginal ulcers in NHL patients. A 31-year-old woman with a history of NHL undergoing R-CHOP (rituximab, cyclophosphamide, doxorubicin, vincristine, and prednisone) chemotherapy regimen was referred with complaints of painful and purulent ulcers in the perianal and vaginal areas. Physical examination showed multiple well-circumscribed ulcers with purulent exudate. Laboratory examinations ruled out STIs, and pathergy test results were positive. Based on the history, clinical findings, and supporting examination results, the patient was diagnosed with genital ulcers due to BD with secondary infection. The diagnosis of genital ulcers in BD is based on clinical criteria and positive pathergy test results, after excluding differential diagnoses such as genital herpes, warts, and syphilis. A history of NHL and chemotherapy likely influenced this patient’s clinical presentation and immune response. Although positive herpes simplex virus (HSV)-2 IgG serology suggested past infection, there was no evidence of acute infection, but HSV reactivation may have been a trigger or aggravating factor in BD lesions. Management included systemic and topical antibiotics to treat secondary infection and local treatments to reduce inflammation and promote healing. This case illustrates an atypical presentation of genital ulcers due to BD in a patient with NHL undergoing chemotherapy. To make the right diagnosis and treat this patient, a thorough diagnostic process involving the patient’s history, physical examination, laboratory work, and pathology testing was necessary.

## Introduction

Behçet’s disease (BD), a type of vasculitis that affects tiny to large blood vessels, is a chronic inflammatory illness that affects many body systems [1]. The prevalence of BD is high in Europe, Asia, and Africa, at 10-420 cases/100,000 population, but is rarely reported in Indonesia. Its pathogenesis involves complex interactions of genetics (human leukocyte antigen (HLA)-B51), infection (herpes simplex virus (HSV), *Streptococcus*), and abnormal immune responses (neutrophil activation, interleukin (IL)-17) [1]. Clinical symptoms include mucosal ulcers in the oral cavity and genitals, skin disorders, uveitis, and involvement of the joints, gastrointestinal tract, nervous system, and vascular system [1]. Compared to the International Study Group criteria, the newly suggested criteria based on multinational data show significantly higher sensitivity while retaining a respectable level of specificity. It is recommended that BD be diagnosed and categorized using the International Criteria for Behçet’s Disease (ICBD) criteria. A patient scoring ≥4 points is classified as having BD [1]. BD was first described in 1937 by Hulusi Behçet as a triad of oral ulcers, genital ulcers, and uveitis. However, not all patients show all three manifestations. The use of the ICBD can help in establishing the diagnosis [2].

Genital ulcers occur in 60-90% of patients with BD, as reported in various studies [1,3]. Genital ulcers in BD are generally recurrent, painful, well-circumscribed, deep, and often leave scarring after healing [4]. This condition can easily be mistaken for a sexually transmitted infection, especially in patients with risk factors that have not been fully explored. The complexity increases when BD occurs in patients with non-Hodgkin lymphoma (NHL), a lymphoid neoplasm that affects humoral and cellular immunity. Studies have shown that patients with lymphoma are at a higher risk for autoimmune manifestations such as BD due to impaired T- and B-cell regulation [5].

This case report discusses a 31-year-old patient with NHL who presented with genital ulceration resembling a sexually transmitted disease, but was later diagnosed with BD. Given atypical genital lesions, this report emphasizes the significance of a thorough diagnostic approach, particularly for patients with intricate immunological comorbidities.

## Case presentation

A 31-year-old woman was referred from the Tulip Integrated Cancer Installation Polyclinic to the Dermatology and Venereology Polyclinic of Dr. Sardjito General Hospital, Yogyakarta, with a chief complaint of painful wounds around the anus and vagina. The patient was diagnosed with NHL in January 2025. The patient had completed four cycles of chemotherapy, with the last cycle administered on April 24, 2025.

About two weeks before being sent to the hospital, the patient reported experiencing constipation, resulting in frequent straining during bowel movements, which was then followed by bleeding from the anus area, followed by the appearance of sores in the mouth area that were painful. About a week later, the complaint developed into a painful wound in the anus and vagina area, accompanied by pus discharge. The patient tried treatment with gentamicin ointment, chloramphenicol, douching with povidone-iodine solution, and topical oil, without any improvement. On the day of the hospital examination, the patient was referred to the Dermatology and Venereology Polyclinic of Dr. Sardjito Hospital from the Tulip section with complaints in the genital area that persisted.

The patient had never experienced similar complaints in the past. The patient was diagnosed with NHL in January 2025. She had undergone the R-CHOP chemotherapy regimen, consisting of a combination of cyclophosphamide, doxorubicin hydrochloride, vincristine sulfate, and prednisone, given every three weeks, and had undergone four cycles. There was no history of trauma or surgery. The patient was diagnosed with BD several weeks ago at the Tulip Clinic. A history of heart disease, high blood pressure, diabetes mellitus, or drug and food allergies was denied.

The patient denied any family history of allergies, history of malignancy, or similar complaints. Regarding personal hygiene and exposure history, the patient had been washing her face and bathing with Lifebuoy®, using gentamicin ointment, chloramphenicol, wiping with povidone-iodine solution, and applying topical oil. The patient was a housewife and married. Risk factors for sexually transmitted infections (STIs) included the patient having attained menarche at the age of 13 years. The patient had her first sexual intercourse at the age of 27 with her husband without using a condom. The total number of sexual partners to date was one person, namely, the husband. The last sexual intercourse was three weeks ago with her husband, and she used a condom. Her sexual orientation was heterosexual and limited to genital contact. The patient sometimes used condoms during sexual intercourse. She denied ever drinking alcohol, using injected drugs, piercing, or using tattoos, but the patient had received a blood transfusion. The patient’s husband also admitted that he had only had sexual intercourse with the patient and had never drunk alcohol, used injected drugs, piercings, tattoos, or received blood transfusions.

**Figure 1:**
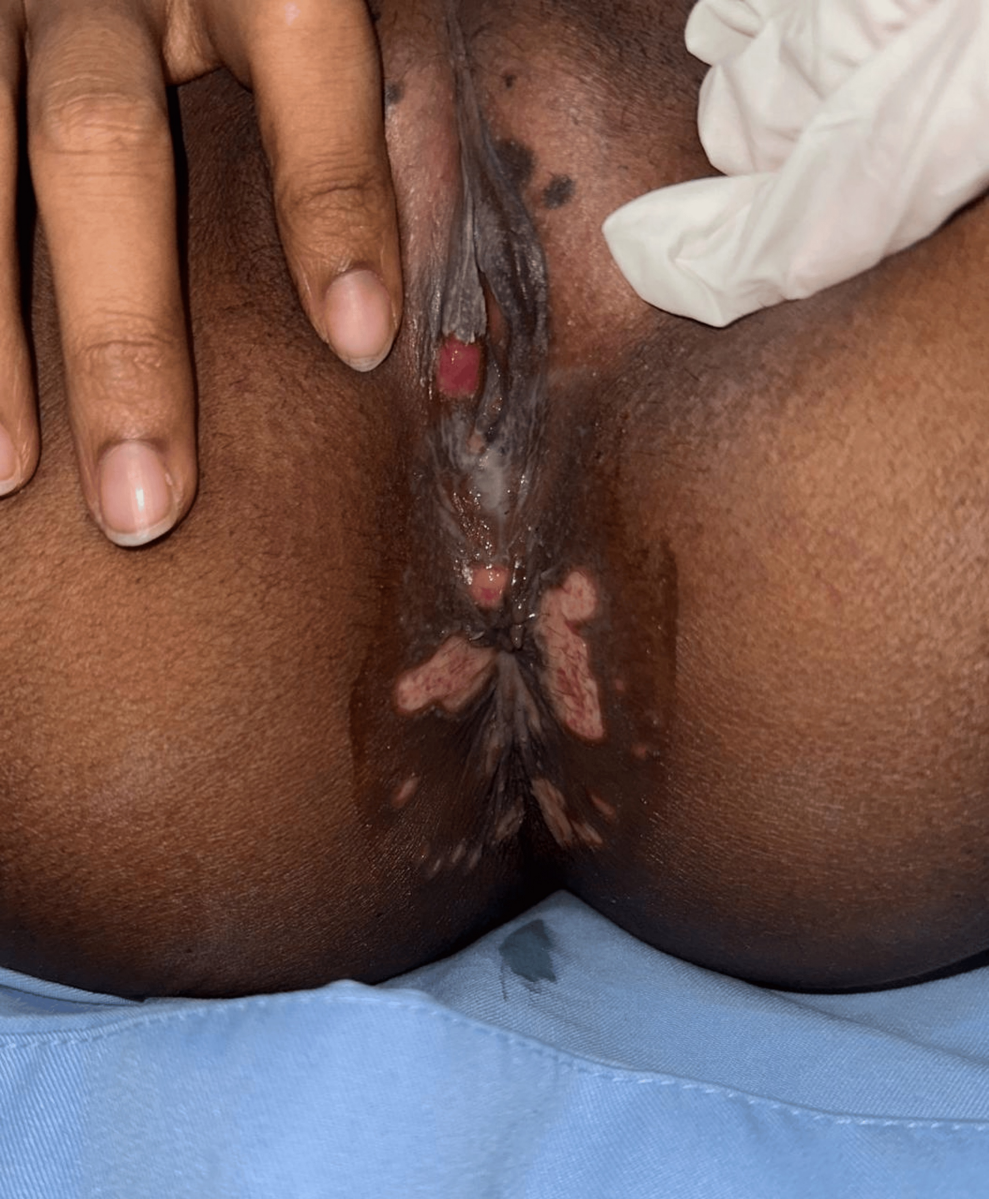
From the perineum to the vaginal vulva, lesions are seen in the form of shallow ulcers and multiple erosions with clear boundaries, accompanied by purulent exudate.

**Figure 2:**
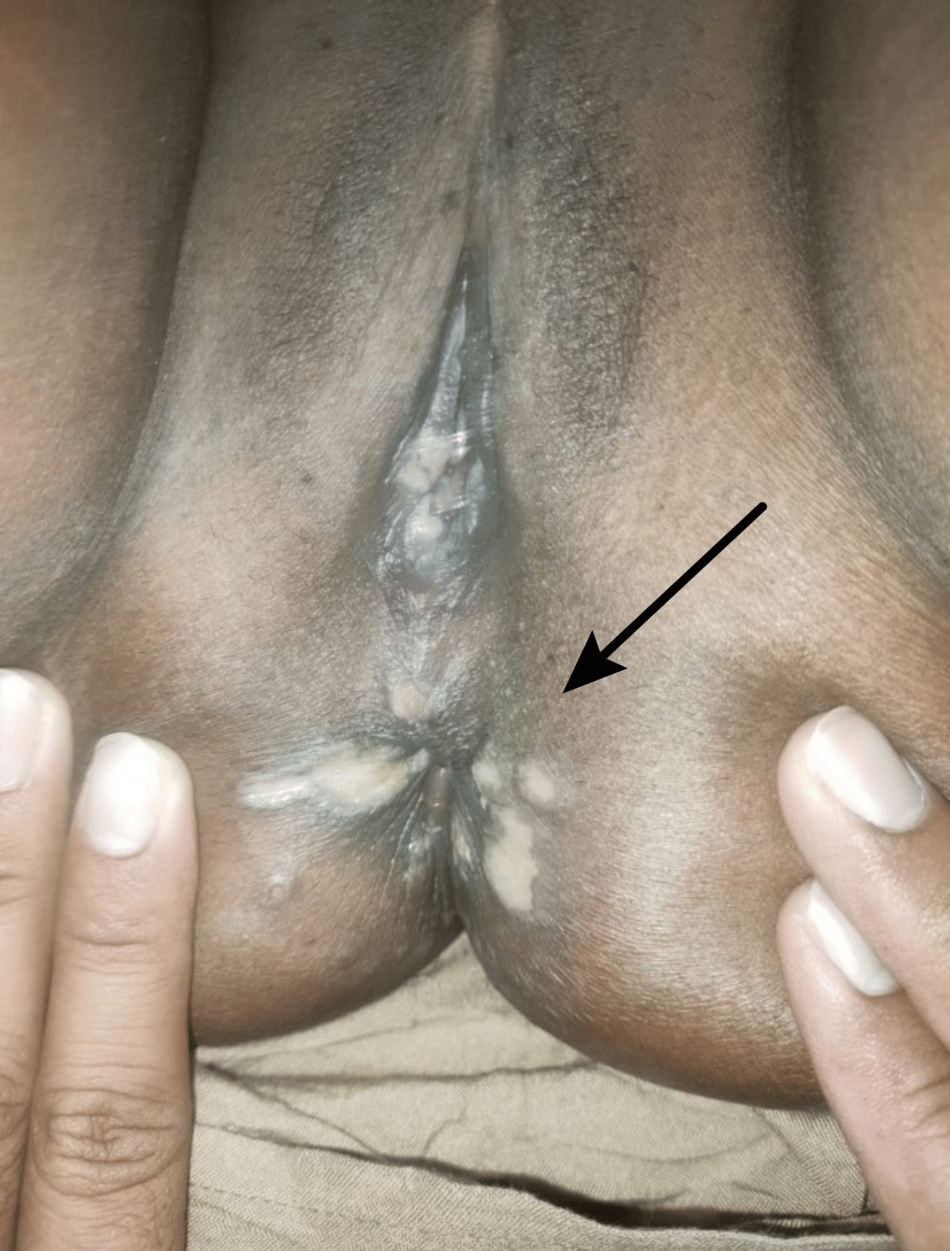
Multiple ulcerative lesions with distinct borders are seen, accompanied by purulent exudate.

On a physical examination, the patient was generally in a compos mentis state, and her vital signs were within normal ranges. There was enlargement of lymph nodes in the cervical area with clear boundaries and soft and mobile consistency, and no tenderness was found on palpation. There were no active verrucous lesions in other external genital areas (Figures 1, 2).

**Figure 3:**
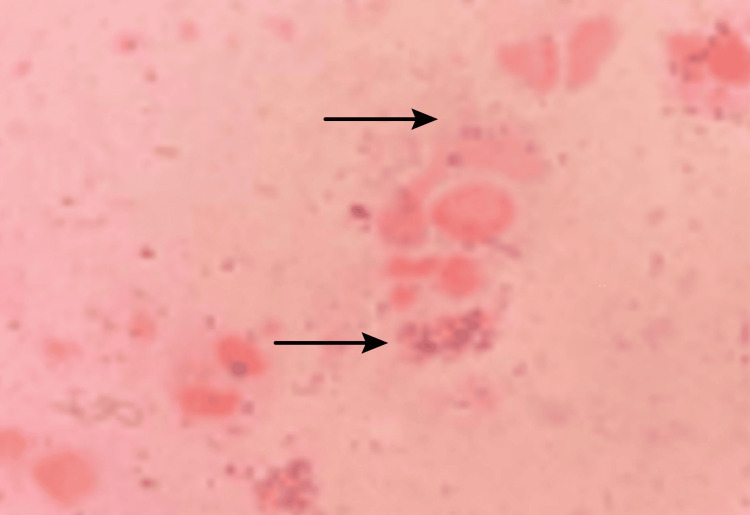
Histological examination of genital ulcers with Gram staining showing the presence of Gram-positive cocci (40×).

For this patient, genital ulcers needed to be further evaluated to determine the underlying etiology. Several possible differential diagnoses that needed to be considered included BD, genital herpes, chancroid ulcers, and syphilis. On laboratory examination, the HIV examination showed non-reactive results (Table 1), while the *Treponema* examination showed negative results (Figure 3).

**Table 1:** Blood test findings. SGOT: serum glutamic-oxaloacetic transaminase; SGPT: serum glutamic-pyruvic transaminase; BUN: blood urea nitrogen; HSV: herpes simplex virus

Test	Results	Reference range	Method
Complete blood count			
Erythrocytes	3.56	4.00–5.40 × 10^6^/μL	Impedance
Hemoglobin	10.3	12.0–15.0 × g/dL	Spectrophotometer
Hematocrit	32.8	35.0–49.0%	Calculation
Leucocytes	7.3	4.50–11.50 × 10^3^/μL	Flow cytometry
Platelets	191	150–450 × 10^3^/μL	Flow cytometry
Liver function			
Albumin	3.92	3.97–4.94 g/dL	Colorimetric assay
SGOT	44	10–35 U/L	Enzymatic colorimetric
SGPT	84	10–35 U/L	Enzymatic colorimetric
Kidney function			
BUN	12	6–20 mg/dL	Urease-GLDH
Creatinine	0.73	0.51–0.95 mg/dL	Jaffe
Diabetes			
Random glucose	94	74–106 mg/dL	Hexokinase
Electrolyte			
Sodium (Na)	131	136–145 mmol/L	ISE
Potassium (K)	4.6	3.5–5.1 mmol/L	ISE
Chloride (Cl)	93	96–107 mmol/L	ISE
Anti-HIV	Non-reactive		
IgM Anti HSV2	0.4	Negative: <20.0 U/mL; borderline: 20.0–25.0 U/mL; positive: >25.0 U/mL	
IgG Anti HSV2	40.7	Negative: <20.0 U/mL; borderline: 20.0–25.0 U/mL; positive: >25.0 U/mL	

The HSV-2 serology examination showed positive IgG anti-HSV-2 with a titer of 40.7 and negative IgM anti-HSV-2, indicating a long-standing infection without evidence of current acute infection. Examination of the lesion smear on the ulcer in the anus and vagina area with Gram stain showed polymorphonuclear cells with positive bacterial cocci. As the patient refused to undergo a speculum insertion due to severe pain in the vaginal area, specimens from the cervix could not be taken for examination.

**Figure 4:**
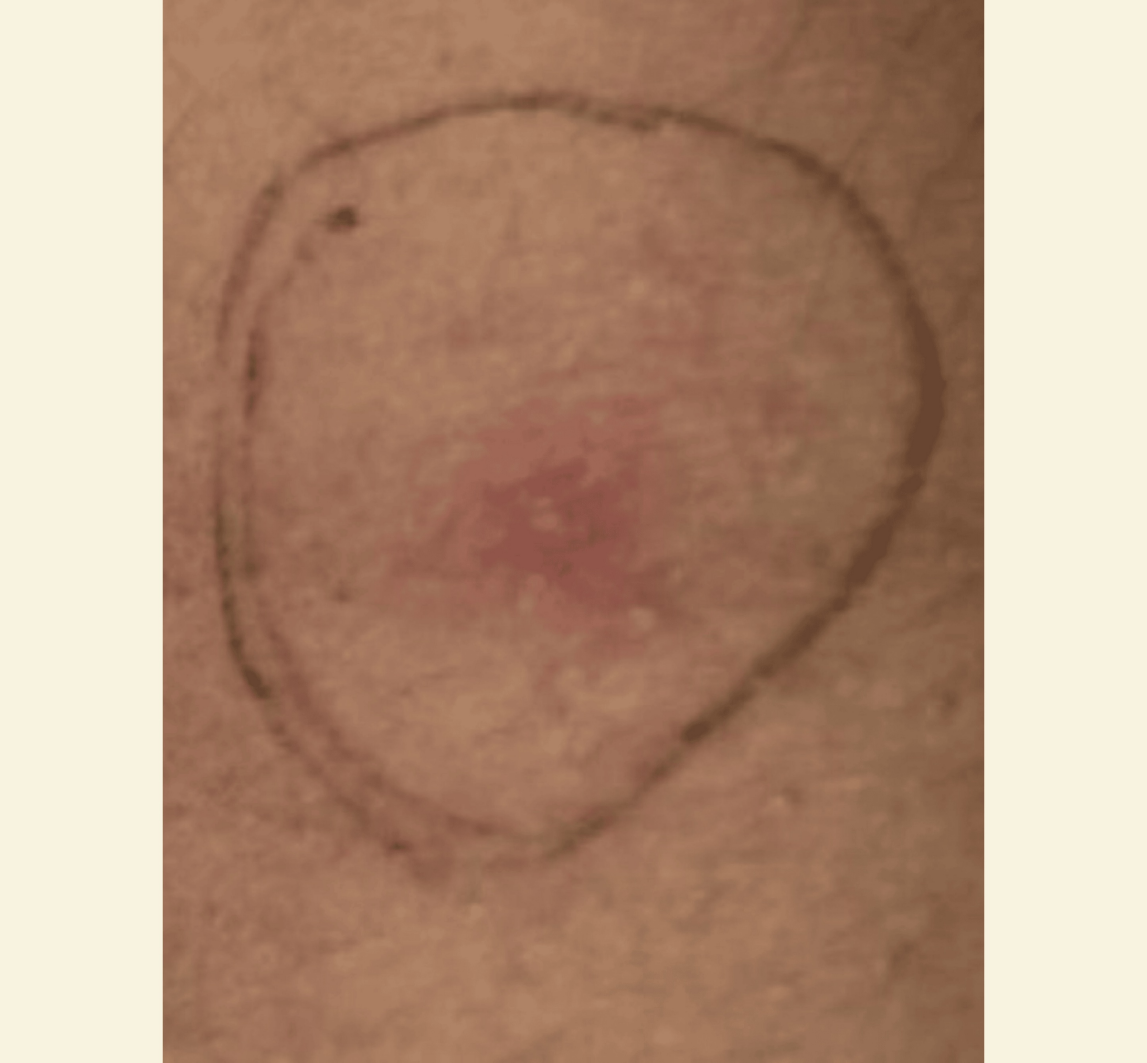
The patient’s pathergy test results indicating a positive reaction, as evidenced by the development of papules larger than 2 mm 48 hours following intradermal injection with a sterile needle.

A diagnostic skin test called the pathergy test evaluates a patient’s heightened inflammatory reaction to mild trauma, such as a needle prick. If a papule (small bump) or pustule (pimple) forms at the prick site within 24 to 48 hours, the test is considered positive. The hypersensitivity of this test makes it a minor diagnostic criterion for BD. The results of the pathergy test in this patient showed a positive reaction, marked by the formation of papules measuring more than 2 mm within 48 hours after intradermal injection using a sterile needle (Figure 4).

Based on the anamnesis, clinical findings on physical examination, and supporting examinations, the patient was diagnosed with genital ulcers, with the most likely etiology being BD, accompanied by secondary infection. The patient received systemic treatment for seven days, which included 100 mg of doxycycline twice a day. As part of local treatment, compresses with 0.9% NaCl solution were applied for 15 minutes twice daily, followed by topical antibiotic mupirocin to the ulcer area. The patient was also educated on maintaining the cleanliness of the genital and perianal regions to support the healing process and prevent recurrent infections.

## Discussion

BD is a chronic, multisystem inflammatory disease characterized by mucosal ulceration in the oral and genital areas, cutaneous lesions, and other systemic manifestations such as uveitis, arthritis, central nervous system involvement, gastrointestinal tract involvement, and vascular involvement [1]. BD is classified as a vasculitis because it can affect blood vessels. Until now, the pathogenesis of BD has not been fully understood. Still, a complex interaction between genetic predisposition (eg, HLA-B51), immune system dysfunction, and environmental factors such as infections is thought to play a role in developing this disease. Although Behçet’s syndrome is characterized by HLA-B51, genetic markers are not very helpful in diagnosing the condition. Instead, it is thought to be a significant determinant of clinical manifestations in this diverse illness [1].

In this case, the patient was a 31-year-old woman with a diagnosis of NHL who underwent the R-CHOP chemotherapy regimen. The patient presented with a chief complaint of painful and purulent ulcers in the perianal and vaginal areas, which were initially suspected to be sexually transmitted infections (STIs), given the clinical presentation that resembled ulcers caused by STIs.

Diagnosing genital ulcers in BD is based on patient history, clinical features, and supporting examinations. From the anamnesis, the main complaint was a persistent painful wound in the area around the anus and vulvovaginal since two weeks before the examination. The complaint did not improve after using various topical ointments and antiseptic solutions. The patient had NHL and had undergone four cycles of chemotherapy with the R-CHOP regimen. There was no history of similar complaints before, and the patient had no history of risky sexual behavior. A dermatologist’s examination showed multiple ulcers with clear boundaries, purulent exudate in the perianal and perineum region, and tenderness and edema. Supporting examinations were performed to exclude differential diagnoses, including STIs. The results of HIV and *Treponema* serology examinations were negative. HSV-2 IgM serology examination had negative results and positive IgG results with a titer of 40.7 and negative IgM, indicating a long-standing infection without evidence of acute illness. Gram smear of the ulcer lesion showed polymorphonuclear cells with Gram-positive cocci, but no bacilli or typical organisms were found. This gave an impression of a secondary infection. Speculum examination could not be performed due to severe pain. Pathergy test showed positive results, with the formation of papules measuring >2 mm within 48 hours after intradermal injection of a sterile needle. This examination was critical to exclude other differential diagnoses of ulcers caused by STIs.

The suggested differential diagnosis was HSV-2-induced vaginal herpes, which is the most common cause of painful recurrent genital ulcers. Herpes ulcers are generally multiple vesicles that quickly turn into shallow ulcers with uneven edges, are painful, and are often accompanied by inguinal lymphadenopathy [6]. In terms of differential diagnosis, genital herpes is an initial consideration, considering the complaints in the form of painful and exudative ulcers that can resemble herpes simplex lesions [7].

However, HSV-2 serology results showed positive IgG with a titer of 40.7 and negative IgM, indicating a past infection with no evidence of current acute infection. However, given that the patient had NHL and underwent chemotherapy, the compromised immune status could have caused HSV reactivation without a detectable IgM response [7]. Therefore, this could not be confirmed as the primary cause. Thus, the possibility of HSV reactivation remains relevant even though IgM was negative. The laboratory findings strengthened the possibility of reactivated latent herpes coinfection, not as the primary cause of the lesions, but as a trigger or comorbidity that worsens the clinical picture of BD [7,8].

Research on the pathophysiology of BD has long focused on the role of viral infections, such as HSV. Research has indicated that HSV can be found in BD patients’ vaginal sores, saliva, and peripheral blood leukocytes [7,8]. This has led to the hypothesis that viral infections, particularly HSV, may act as triggers or exacerbating factors for the inflammatory process in BD [7,8].

The subsequent differential diagnosis was a chancroid ulcer. Chancroid ulcers tend to be single or few, have irregular borders, a necrotic base, bleed easily, and usually do not leave scars [9]. These ulcers are also often accompanied by painful unilateral inguinal lymphadenopathy and can become suppurative (bubo), while in this patient, these signs were not found [9]. In addition, chancroid ulcers usually arise acutely several days to weeks after risky sexual intercourse. At the same time, this patient had no history of recent risky sexual intercourse and showed a history of recurrent lesions and a positive pathergy test that supported the diagnosis of BD [9]. Gram stain examination did not show any Gram-negative coccobacillus typical of chancroid ulcers (*Haemophilus ducreyi*), and culture did not show a specific pathogen [9]. Thus, based on the morphology of the lesion, accompanying symptoms, and supporting examination findings, the diagnosis of chancroid ulcer was excluded.

Another proposed differential diagnosis was a syphilitic ulcer. This type of ulcer is usually solitary, has a clear border, a clean base, is painless, and is not accompanied by purulent exudate [10]. Syphilitic ulcers are also often accompanied by painless regional lymphadenopathy [10]. In this case, the lesions were multiple, very painful, accompanied by purulent exudate, and showed signs of active inflammation, such as edema and significant tenderness, which are not commonly found in primary syphilis [10]. In addition, supporting examinations in this patient also did not support the diagnosis of syphilis. Moreover, the results of the *Treponema* test (possibly *Treponema pallidum* hemagglutination or Venereal Disease Research Laboratory) showed negative results. The symptoms further strengthened the fact that syphilis was not the cause of ulcers in this patient. Therefore, both from the clinical aspect and laboratory results, the diagnosis of syphilis was ruled out and was more supportive of BD as the primary etiology of ulcers.

BD has no specific test that can definitively identify it. Information from clinically confirmed BD patients and controls who exhibited at least one significant symptom of BD or a BD-like disorder was collected and submitted to an international team. This team reviewed the BD criteria to establish internationally recognized guidelines. As a result, the ICBD criteria were developed [11]. Genital ulcers in BD are reported to occur in 60-90% of cases and are usually painful, deep, well-circumscribed, recurrent, and may leave scars [11]. In this patient, multiple ulcers with purulent exudate and significant tenderness suggested the possibility of secondary infection, which can worsen the course of the disease and mask the typical characteristics of BD ulcers. The diagnosis of BD is based on a combination of clinical findings and classification criteria. The ICBD criteria, which include recurrent oral ulcers, genital ulcers, skin lesions, uveitis, neurologic involvement, vascular, and pathergy testing, were used as a diagnostic guideline in this case (Table 2) [11].

**Table 2:** International Criteria for Behçet’s Disease (ICBD). Reproduced from Marfatia et al. [11] licensed under Creative Commons.

Symptom	Point
Ocular lesion (recurrent)	2
Oral aphtosis (recurrent)	2
Genital aphtosis (recurrent)	2
Skin lesions (recurrent)	1
Central nervous system	1
Vascular manifestations	1
Positive pathergy test	1
Behcet’s disease score of ≥4 indicates Behcet’s disease	

In this case, the patient had multiple painful oral ulcers (2 points), multiple painful genital ulcers (2 points), and a positive pathergy test result (1 point), with a total score of 5 points. Hence, BD was established from the assessment criteria. Although there were no skin lesions such as erythema nodosum or papulopustular eruption, the combination of two typical mucosal manifestations and a positive pathergy test was strong enough to establish the diagnosis of BD. The signs of disease show the importance of considering the variation in clinical presentation of BD, which does not always involve the entire spectrum of symptoms.

BD is uncommon in the general population, which is significant. However, in immunocompromised patients, like this patient with NHL who received chemotherapy, it can happen or recur [5]. In this patient, the use of the R-CHOP chemotherapy regimen may cause T/B-cell dysregulation, triggering BD flares through increased proinflammatory cytokines (e.g., IL-17, tumor necrosis factor-α) [5]. Immunosuppression may trigger immune dysregulation, reactivation of autoimmune disease, or worsen mucocutaneous manifestations [5]. In addition, there is evidence that viral infections such as HSV can trigger activation of the innate immune system and T cells that play a role in the pathogenesis of BD. The sign of disease opens up the possibility of a bidirectional relationship between BD and HSV as a co-infection or trigger for BD exacerbations [8].

Management of genital ulcers in BD focuses on relieving pain, controlling local inflammation, accelerating lesion healing, and preventing recurrence [12]. For mild-to-moderate ulcers, topical corticosteroids such as clobetasol propionate 0.05% are the primary choice because they effectively suppress local inflammation and accelerate healing [12]. If there are signs of secondary infection, topical antibiotics such as mupirocin or gentamicin can help treat superficial infections [12].

In this case, the therapy chosen combined topical and systemic treatments. The patient was given NaCl compresses, topical mupirocin to treat possible secondary infections, and oral doxycycline. Doxycycline was chosen not only for its antibacterial effect but also because it has a mild anti-inflammatory effect that can help reduce mucosal inflammation. In addition, its good safety profile makes it a rational choice for patients with immunocompromised status after chemotherapy [12]. This approach aims to control local inflammation without overloading the immune system while evaluating the patient’s clinical response to first-line therapy.

## Conclusions

This case confirms that BD can present with atypical genital ulcers in immunocompromised patients, such as those with NHL. A combination of typical symptoms, positive pathergy tests, and exclusion of differential diagnoses confirmed the diagnosis. HSV-2 infection may have been a trigger, but it was not the primary cause. Therapy was tailored to the patient’s immune status, emphasizing control of inflammation and prevention of secondary infections.

## References

[1] Bulur I, Onder M (2017). Behçet disease: new aspects. Clin Dermatol.

[2] Nair JR, Moots RJ (2017). Behcet's disease. Clin Med (Lond).

[3] Mat MC, Goksugur N, Engin B, Yurdakul S, Yazici H (2006). The frequency of scarring after genital ulcers in Behçet's syndrome: a prospective study. Int J Dermatol.

[4] Obata S, Kobayashi K, Toda M, Miyagi E, Aoki S (2019). Genital ulcer of Behçet disease localized in the vagina may lack pain, making it difficult to assess. Case Rep Rheumatol.

[5] Aya A, Dawson A, Patel P, Acosta CL, Dedona A (2022). Rapid progression of large B-cell lymphoma in Behçet's disease on immunosuppressive therapy: a case report with literature review. Cureus.

[6] Kaneko F, Togashi A, Saito S (2011). Behçet's disease (Adamantiades-Behçet's disease). Clin Dev Immunol.

[7] Chang A, Sholukh AM, Wieland A (2022). Herpes simplex virus lymphadenitis is associated with tumor reduction in a patient with chronic lymphocytic leukemia. J Clin Invest.

[8] Sohn S, Lee ES, Bang D, Lee S (1998). Behçet's disease-like symptoms induced by the herpes simplex virus in ICR mice. Eur J Dermatol.

[9] Ndzomo P, Deghmane AE, Tchatchouang S (2025). Genetic characterization of Haemophilus ducreyi from non-genital skin lesions in Cameroon. J Infect.

[10] Shmaefsky B, Alcamo IE, and Heymann DL (2009). Syphilis: Deadly Diseases and Epidemics Series. https://books.google.co.id/books?id=8UDhgXbMpuYC&printsec=frontcover&hl=id&source=gbs_ge_summary_r&cad=0#v=onepage&q&f=false.

[11] Marfatia YS, Patel HK, Mahajan R, Ninama K (2020). Behcet disease - a nonvenereal cause of genital ulceration. Indian J Sex Transm Dis AIDS.

[12] Aldeen T (2009). Behçet's disease, painful genital ulcerations and steroid pulse therapy. BMJ Case Rep.

